# Supplementary material for: The Evolution and Characterization of the RNA Interference Pathways in Lophotrochozoa
Source: Genome Biol Evol. 2024 May 7;16(5):evae098. doi: 10.1093/gbe/evae098 (PMC11114477; doi:10.1093/gbe/evae098)
Supplement: evae098_Supplementary_Data [file evae098_supplementary_data.zip › Supplementary_materials.zip!/selected_miRNAs/NoGe_selected_miRNAs/mirdeep_pdf_files/NMRB01000795.1_Notospermus_geniculatus_strain_Ushimado_scaffold795,_whole_genome_shotgun_sequence_20947.pdf]

Provisional ID	:	NMRB01000795.1_Notospermus_geniculatus_strain_Ushimado_scaffold795,_whole_genome_shotgun_sequence_20947
Score total	:	2573.2
Score for star read(s)	:	3.9
Score for read counts	:	2569.1
Score for mfe	:	0.8
Score for randfold	:	0
Score for cons. seed	:	-0.6
Total read count	:	5051
Mature read count	:	4941
Loop read count	:	0
Star read count	:	110

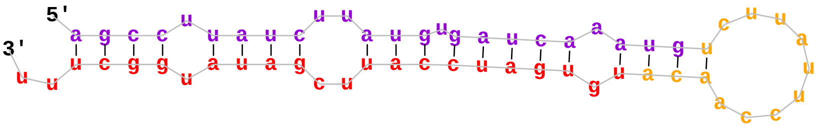

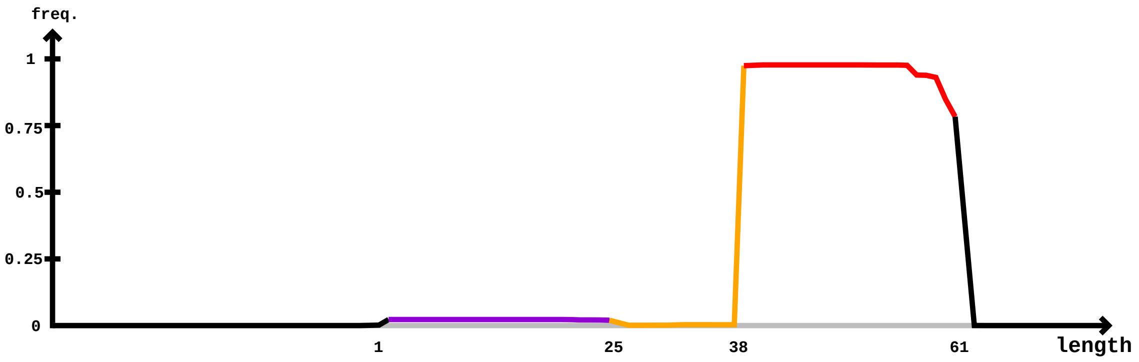


```
                                         Star                                       Mature
5'- acugguuuggucucugacagggaugaguacugcagccuuaucuuaugugaucaaaugucuuauuccaacaugugauccauucgauauggcuuugguacaaacuccaaa   -3'   obs
    acugguuuggucucugacagggaugaguacugcagccuuaucuuaugugaucaaaugucuuauuccaacaugugauccauucgauauggcuuugguacaaacuccaaa         exp
    .......(.((((((...)))))).)((((((.((((.((((..(((.(((((.((((.........)))).))))))))..)))).)))).))))))..........   reads   mm   sample
    .................................agccuuaucuuaugugaucaaau....................................................   1       0    Mm3
    .................................agccuuaucuuaugugGucaaaug...................................................   3       1    Mm3
    .................................agccuuaucuuaugugaGcaaaug...................................................   1       1    Mm3
    .................................agccuuaucuuaugugaucaaaug...................................................   15      0    Mm3
    .................................agccuuaucuuaugugCucaaaug...................................................   1       1    Mm3
    .................................agccuuaucuuaugugaucaaaugu..................................................   2       0    Mm3
    .................................agccuuaucuuaugugaucaaaugucuuauuccaaca......................................   1       0    Mm3
    .........................................................ucuuauuccaacaugugauccauucg.........................   1       0    Mm3
    ......................................................................ugugauccauucgauaug....................   19      0    Mm3
    ......................................................................ugugauccauucgauauU....................   1       1    Mm3
    ......................................................................ugugauccauucgauauA....................   1       1    Mm3
    ......................................................................ugugauccauucgauaugg...................   1       0    Mm3
    ......................................................................ugugauccauCcgauauggc..................   1       1    Mm3
    ......................................................................ugugauccauucgauauggc..................   4       0    Mm3
    ......................................................................ugugauccauAcgauauggcu.................   1       1    Mm3
    ......................................................................ugugauccauucgauauAgcu.................   1       1    Mm3
    ......................................................................ugugauccauucgauauggcu.................   49      0    Mm3
    ......................................................................ugugauccUuucgauauggcu.................   1       1    Mm3
    ......................................................................ugugauccauucgauauggcuu................   26      0    Mm3
    ......................................................................ugugauccauucgauauAgcuu................   1       1    Mm3
    ......................................................................ugugauccauucgauauggcuuA...............   4       1    Mm3
    ......................................................................ugugauccUuucgauauggcuuu...............   1       1    Mm3
    ......................................................................ugugauccauGcgauauggcuuu...............   1       1    Mm3
    ......................................................................ugugauccauucgauauAgcuuu...............   2       1    Mm3
    ......................................................................ugugauccauucgauauggcuuu...............   434     0    Mm3
    ......................................................................ugugauccauAcgauauggcuuu...............   2       1    Mm3
    ......................................................................ugugauccauCcgauauggcuuu...............   3       1    Mm3
    ......................................................................ugugauccauucgauaAggcuuu...............   1       1    Mm3
    ......................................................................ugugauccGuucgauauggcuuu...............   1       1    Mm3
    ......................................................................ugugauccauucgauGuggcuuu...............   1       1    Mm3
    ......................................................................ugugauccauucgauaCggcuuu...............   2       1    Mm3
    ......................................................................ugugauccauucgauauCgcuuu...............   1       1    Mm3
    ......................................................................ugugauccauucgauauUgcuuu...............   2       1    Mm3
```

```
                                Star                                 Mature
acugguuuggucucugacagggaugaguacugcagccuuaucuuaugugaucaaaugucuuauuccaacaugugauccauucgauauggcuuugguacaaacuccaaa
......................................................................ugugauccauucgauauggcuuuA..............   3     1     Mm3
......................................................................ugugauccauucgauauggcuuuU..............   14    1     Mm3
......................................................................ugugauccauucgauauggcuuuC..............   2     1     Mm3
........................................................................ugauccauucgauauggcuuu...............   1     0     Mm3

.................................agccuuaucuuaugugaucaaaug...................................................   3     0     Fm3
......................................................................ugugauccauucgauauggcu.................   10    0     Fm3
......................................................................ugugauccauucgauauggcuuu...............   2     0     Fm3

................................cagccuuaucuuaugugauca.......................................................   3     0     Mm1
.................................agccuuaucuuaugugaucaaaug...................................................   11    0     Mm1
...............................................................uccaacaugugauccauucgauau.....................   5     0     Mm1
......................................................................ugugUuccauucgauaug....................   1     1     Mm1
......................................................................uCugauccauucgauaug....................   1     1     Mm1
......................................................................ugugauccauucgauauU....................   1     1     Mm1
......................................................................ugugauccauucgauaAg....................   1     1     Mm1
......................................................................ugugauccauucgauaGg....................   2     1     Mm1
......................................................................ugugauccauucgauUug....................   1     1     Mm1
......................................................................uAugauccauucgauaug....................   1     1     Mm1
......................................................................ugugauccauucgauaug....................   110   0     Mm1
......................................................................ugugauccauucgauauggc..................   16    0     Mm1
......................................................................ugugauccauucgauauggcG.................   4     1     Mm1
......................................................................ugugauccauucgauauggcu.................   174   0     Mm1
......................................................................ugugauccauucgauauggcC.................   1     1     Mm1
......................................................................ugugauccauucgauaCggcu.................   1     1     Mm1
......................................................................ugugauccauuUgauauggcu.................   1     1     Mm1
......................................................................ugugauccUuucgauauggcu.................   1     1     Mm1
......................................................................ugugauccauucgauauCgcu.................   1     1     Mm1
......................................................................ugugauccauucgauauAgcu.................   1     1     Mm1
......................................................................ugugauccauucgauaCggcuu................   2     1     Mm1
......................................................................ugugauccauucgauauUgcuu................   1     1     Mm1
......................................................................ugugauccauucgauauggcuu................   129   0     Mm1
......................................................................ugugauccauGcgauauggcuuu...............   3     1     Mm1
......................................................................ugugauccauucgauauggcuuA...............   20    1     Mm1
......................................................................ugugauUcauucgauauggcuuu...............   2     1     Mm1
......................................................................ugugauccauucgauauCgcuuu...............   6     1     Mm1
......................................................................ugugUuccauucgauauggcuuu...............   1     1     Mm1
......................................................................ugugauccauucgauaGggcuuu...............   4     1     Mm1
......................................................................ugugauccauuUgauauggcuuu...............   1     1     Mm1
......................................................................ugugauccauucgauauggcCuu...............   1     1     Mm1
......................................................................ugugauccauucgauaAggcuuu...............   4     1     Mm1
......................................................................ugugauccGuucgauauggcuuu...............   1     1     Mm1
......................................................................Cgugauccauucgauauggcuuu...............   1     1     Mm1
......................................................................ugugauccauAcgauauggcuuu...............   3     1     Mm1
......................................................................ugugauccauucgauauggcuuu...............   971   0     Mm1
......................................................................ugugauccauucgauaCggcuuu...............   6     1     Mm1
......................................................................ugugauccauucgauauUgcuuu...............   6     1     Mm1
......................................................................ugugauccauucgauauAgcuuu...............   4     1     Mm1
......................................................................ugugauccauucgauauggcuuuA..............   11    1     Mm1
......................................................................ugugauccauucgauauggcuuug..............   2     0     Mm1
......................................................................ugugauccauucgauauggcuuuU..............   8     1     Mm1

...............................gcagccuuaucuuaugugauc........................................................   2     0     Mm2
...............................gcagccuuaucuuaugugauca.......................................................   2     0     Mm2
................................cagccuuaucuuaugugauca.......................................................   1     0     Mm2
................................cagccuuaucuuaugugCucaaa.....................................................   1     1     Mm2
.................................agccuuaucuuaugugaucaaau....................................................   4     0     Mm2
.................................agccuuaucuuaugugaucaaaug...................................................   60    0     Mm2
.................................agccuuaucuuaugugaucaaaugucuuauucca.........................................   1     0     Mm2
.................................agccuuaucuuaugugaucaaaugucuuauuccaaca......................................   3     0     Mm2
.............................................................auuccaacauUugauccauucga.........................   1     1     Mm2
...............................................................uccaacaugugauccauucgauaug....................   1     0     Mm2
................................................................ccaacaugugauccauucgauaug....................   2     0     Mm2
................................................................ccaacaugugauccauucgauauggcuuu...............   1     0     Mm2
......................................................................ugugauccauucgauaug....................   42    0     Mm2
......................................................................ugugauccauucgauauC....................   1     1     Mm2
......................................................................ugugauccauucgauaugg...................   2     0     Mm2
......................................................................ugugauccauucgauaugU...................   2     1     Mm2
......................................................................ugugauccauucgauauggA..................   1     1     Mm2
......................................................................ugugauccauucgauauggc..................   18    0     Mm2
```

Star Mature

```
acugguuuggucucugacagggaugaguacugcagccuuaucuuaugugaucaaaugucuuauuccaacaugugauccauucgauauggcuuugguacaaacuccaaa
......................................................................ugugauccauucgauauUgcu.................   1     1   Mm2
......................................................................ugugauccauucgauauggcu.................   160   0   Mm2
......................................................................ugugauccauucgauauggcG.................   4     1   Mm2
......................................................................ugugaAccauucgauauggcu.................   1     1   Mm2
......................................................................ugugauccauucgauauCgcu.................   1     1   Mm2
......................................................................ugugauccauucgauaAggcu.................   2     1   Mm2
......................................................................ugugauccauucgauauggcCu................   1     1   Mm2
......................................................................ugugauccauucgauauCgcuu................   1     1   Mm2
......................................................................ugugauccauucAauauggcuu................   1     1   Mm2
......................................................................ugugauccauucgauaGggcuu................   1     1   Mm2
......................................................................ugugauccauucgauauggcuG................   1     1   Mm2
......................................................................uguCauccauucgauauggcuu................   1     1   Mm2
......................................................................uguAauccauucgauauggcuu................   2     1   Mm2
......................................................................ugugauccauucgauaAggcuu................   1     1   Mm2
......................................................................ugugauccauucgauauggcuu................   162   0   Mm2
......................................................................ugugauccauucgauaugCcuuu...............   1     1   Mm2
......................................................................ugugauccauGcgauauggcuuu...............   2     1   Mm2
......................................................................ugugauccauucgauGuggcuuu...............   2     1   Mm2
......................................................................ugugauccUuucgauauggcuuu...............   1     1   Mm2
......................................................................ugugauccauucgauauggcuuG...............   2     1   Mm2
......................................................................ugugauccauucAauauggcuuu...............   1     1   Mm2
......................................................................ugugauccauCcgauauggcuuu...............   1     1   Mm2
......................................................................ugugaAccauucgauauggcuuu...............   1     1   Mm2
......................................................................uguAauccauucgauauggcuuu...............   1     1   Mm2
......................................................................ugugauccauucgauauggcuuC...............   1     1   Mm2
......................................................................ugugauccauucgaGauggcuuu...............   1     1   Mm2
......................................................................ugGgauccauucgauauggcuuu...............   1     1   Mm2
......................................................................ugugauccCuucgauauggcuuu...............   1     1   Mm2
......................................................................ugugauccauucgauCuggcuuu...............   1     1   Mm2
......................................................................ugugauAcauucgauauggcuuu...............   1     1   Mm2
......................................................................ugugauccGuucgauauggcuuu...............   1     1   Mm2
......................................................................ugugaucUauucgauauggcuuu...............   1     1   Mm2
......................................................................ugugauccauucgauaGggcuuu...............   2     1   Mm2
......................................................................ugugauccauucgauauggcuAu...............   1     1   Mm2
......................................................................ugugauccauAcgauauggcuuu...............   5     1   Mm2
......................................................................ugCgauccauucgauauggcuuu...............   2     1   Mm2
......................................................................ugugCuccauucgauauggcuuu...............   1     1   Mm2
......................................................................ugugauccaCucgauauggcuuu...............   1     1   Mm2
......................................................................Cgugauccauucgauauggcuuu...............   3     1   Mm2
......................................................................ugugauccauucgauaugUcuuu...............   2     1   Mm2
......................................................................ugugauccauucgauauggcCuu...............   3     1   Mm2
......................................................................ugugauccauucgauauUgcuuu...............   10    1   Mm2
......................................................................ugugauccauucgauauggcuuu...............   2216  0   Mm2
......................................................................ugugauccauucgauauggcuuA...............   1     1   Mm2
......................................................................ugugauccauucUauauggcuuu...............   1     1   Mm2
......................................................................ugAgauccauucgauauggcuuu...............   1     1   Mm2
......................................................................ugugauccauucgauaAggcuuu...............   5     1   Mm2
......................................................................ugugUuccauucgauauggcuuu...............   1     1   Mm2
......................................................................ugugauccauucgauauggUuuu...............   1     1   Mm2
......................................................................ugugauccauucgauaCggcuuu...............   8     1   Mm2
......................................................................ugugauccauucgauauCgcuuu...............   5     1   Mm2
......................................................................ugugauccauucgauauAgcuuu...............   13    1   Mm2
......................................................................ugugauccauucgauauggcuuuA..............   4     1   Mm2
......................................................................ugugauccauucgauauggcuuuC..............   1     1   Mm2
......................................................................ugugauccauucgauauggcuuuU..............   88    1   Mm2
.......................................................................gugauccauucgauauggcuuu...............   6     0   Mm2
.......................................................................gugauccauucgauauUgcuuu...............   1     1   Mm2
........................................................................ugauccauucgauauggcuuu...............   5     0   Mm2
........................................................................ugauccauucgauauggcuuugg.............   1     0   Mm2

......................................................................ugugauccauucgauauUgcuuu...............   2     1   Fm2
......................................................................ugugauccauucgauauggcuuu...............   13    0   Fm2
......................................................................ugugauccUuucgauauggcuuu...............   1     1   Fm2
......................................................................ugugauccauucgauaGggcuuu...............   1     1   Fm2

......................................................................ugugauccauucgauauggcuu................   2     0   Fm1
......................................................................ugugauccauucgauauggcuuu...............   4     0   Fm1
......................................................................ugugauccauucgauauggcuuuU..............   6     1   Fm1
```
